# Complete Genomic Sequence of Pseudoalteromonas sp. Strain SAO4-4, a Protease-Producing Bacterium Isolated from Seawater of the Atlantic Ocean

**DOI:** 10.1128/genomeA.00284-18

**Published:** 2018-05-31

**Authors:** Bai-Lu Tang, Jin-Cheng Rong, Yan-Ru Dang, Bin-Bin Xie, Xiu-Lan Chen, Xi-Ying Zhang

**Affiliations:** a State Key Laboratory of Microbial Technology, Marine Biotechnology Research Center, Shandong University, Jinan, China

## Abstract

The complete genome of Pseudoalteromonas sp. strain SAO4-4, a protease-producing bacterium from seawater, is composed of two circular chromosomes and one plasmid. This genome sequence will provide a better understanding of the ecological roles of protease-producing bacteria in the degradation of organic matter in marine aquatic environments.

## GENOME ANNOUNCEMENT

A protease-producing bacterium, Pseudoalteromonas sp. strain SAO4-4, was isolated from seawater of the Atlantic Ocean. The total genomic DNA was extracted using a PowerMax soil DNA isolation kit (Mo Bio Laboratories, Carlsbad, CA, USA). The complete genome sequence of strain SAO4-4 was determined using a combination of PacBio RS II single-molecule real-time (SMRT) sequencing technology (Pacific Biosciences, Menlo Park, CA, USA) and Solexa sequencing (MiSeq platform; Illumina, USA). A total of 808 Mb of clean data (6,466,304 reads) from the Solexa sequencing were obtained, representing 162-fold coverage. *De novo* assembly was performed using the Hierarchical Genome Assembly Process (HGAP) pipeline of the SMRT Analysis version 2.3.0. A total of 3 contigs greater than 500 bp were obtained. Gene calling and annotation were performed using Rapid Annotations using Subsystems Technology (RAST) version 2.0 (1). The signal peptide was predicted using SignalP 4.0 (2). The *MEROPS* categories of peptidases were determined by a BLAST search against the *MEROPS* database (3).

The genome of strain SAO4-4 comprises two circular chromosomes (1 and 2) and one plasmid, having a total size of 5,099,119 bp, with a mean G+C content of 40.7 mol%. Chromosomes 1 and 2 contain 3,282,406 bp and 1,667,761 bp, respectively, and have similar G+C contents (41.0 mol% and 40.4 mol%, respectively) and percentages of coding regions (90.1% and 87.3%, respectively). The genome in total has 4,556 protein-coding genes and 122 RNA genes.

The genome of strain SAO4-4 encodes 90 peptidases having signal peptides, and these predicted peptidases belong to five different peptidase types. Eighty-three of these 90 secreted peptidases (92%) are serine peptidases and metallopeptidases. These 83 serine peptidases and metallopeptidases can be assigned into 19 metallopeptidase families and 10 serine peptidase families listed in the *MEROPS* peptidase database (3). Meanwhile, strain SAO4-4 contains many predicted extracellular enzymes able to hydrolyze oligosaccharides and two enzymes able to hydrolyze phospholipids, which are the major structural components of the cell membrane that constitute significant fractions of organic phosphorus in marine dissolved and particulate organic matter (4). Strain SAO4-4 harbors genes coding for four chitinases, two amylases, one glucanase, and one xylanase, implying that the strain may degrade different kinds of polysaccharides. The strain also contains one predicted extracellular DNase, which indicates that it is capable of utilizing extracellular DNAs ubiquitous in seawater and sediments (5) as nutrient (carbon, nitrogen, and phosphorus) sources.

In summary, the genome analysis for strain SAO4-4 identifies many genes coding for extracellular enzymes able to decompose protein and other polymeric organic substances, including polysaccharides, lipids, and DNA, providing a better understanding of the ecological roles of the strain and its extracellular enzymes, especially proteases, in the degradation of organic matter and elemental cycles (carbon, nitrogen, and phosphorus) in seawater.

### Accession number(s).

The complete genome sequence of Pseudoalteromonas sp. SAO4-4 has been deposited in DDBJ/EMBL/GenBank under the accession numbers CP023398 for chromosome 1, CP023399 for chromosome 2, and CP023400 for plasmid pl.
